# Virologic Tools for HCV Drug Resistance Testing

**DOI:** 10.3390/v7122941

**Published:** 2015-12-04

**Authors:** Slim Fourati, Jean-Michel Pawlotsky

**Affiliations:** National Reference Center for Viral Hepatitis B, C, and D; Department of Virology, Hôpital Henri Mondor, Université Paris-Est and INSERM U955, Créteil 94010, France; jean-michel.pawlotsky@aphp.fr

**Keywords:** HCV, RAVs, resistance, tools, direct-acting antivirals

## Abstract

Recent advances in molecular biology have led to the development of new antiviral drugs that target specific steps of the Hepatitis C Virus (HCV) lifecycle. These drugs, collectively termed direct-acting antivirals (DAAs), include non-structural (NS) HCV protein inhibitors, NS3/4A protease inhibitors, NS5B RNA-dependent RNA polymerase inhibitors (nucleotide analogues and non-nucleoside inhibitors), and NS5A inhibitors. Due to the high genetic variability of HCV, the outcome of DAA-based therapies may be altered by the selection of amino-acid substitutions located within the targeted proteins, which affect viral susceptibility to the administered compounds. At the drug developmental stage, preclinical and clinical characterization of HCV resistance to new drugs in development is mandatory. In the clinical setting, accurate diagnostic tools have become available to monitor drug resistance in patients who receive treatment with DAAs. In this review, we describe tools available to investigate drug resistance in preclinical studies, clinical trials and clinical practice.

## 1. Introduction

Hepatitis C virus (HCV) is a positive-polarity, single-stranded RNA virus belonging to the *Flaviviridae* family, genus *Hepacivirus*. Over 150 million individuals are persistently infected by HCV worldwide, and at risk of developing advanced liver disease and hepatocellular carcinoma [1,2].

Over the past decade, the development of novel molecules that target specific steps of the HCV lifecycle has significantly expanded the “pool” of antiviral drugs available for chronic hepatitis C treatment. The advent of interferon-free combination regimens with “direct-acting antiviral” (DAA) drugs comes with high expectations for very highly sustained virological response (SVR) rates and ultimate control of the epidemics [3,4]. Currently available HCV DAAs are classified into four categories on the basis of their molecular target and mechanism of action: NS3/4A protease inhibitors, NS5A inhibitors, nucleotide analogue inhibitors of NS5B RNA-dependent RNA polymerase (RdRp), and non-nucleoside inhibitors of RdRp. Although SVR rates are very high (over 90%) with the new interferon-free regimens, clinicians will be faced in the future with the potential for treatment failures due to selection of DAA-resistant viruses.

HCV is prone to develop resistance to DAAs, as a result of the lack of proof-reading activity of the viral RdRp coupled with the highly replicative nature of HCV. This leads to the daily generation of large numbers of genetically distinct viral variants within each infected individual [5,6]. The different isolates generated daily form, as a result of natural selection, a unique “quasispecies”. Some quasispecies variants bear polymorphisms in drug-targeted genes, some of which confer reduced susceptibility to DAAs [7]. The prevalence of intrinsically resistant variants within a patient’s quasispecies is in part determined by their replicative fitness and the selective advantages they bear compared with the other viral populations. Typically, a dominant variant is detectable within the viral quasispecies along with less fit variants present at lower frequencies. The presence of minor populations of resistance-associated variants (RAVs) at the start of treatment may affect the outcome of antiviral therapy, because they can become dominant in the context of the selective pressure exerted by the drugs, subsequently leading to a virological breakthrough during treatment or, more frequently with interferon-free regimens, a relapse after treatment cessation [8]. In addition, some RAVs (in particular those conferring resistance to NS5A inhibitors) are very fit and thus persist as the dominant species for months to years after treatment cessation.

Understanding drug resistance is important in the clinical setting in order to optimize treatment regimens, increase success rates and minimize the impact of treatment failure. In this review, we describe tools available to investigate HCV DAA resistance in preclinical and clinical research as well as in clinical practice.

## 2. Virologic Tools Used at the Preclinical Developmental Stage

Candidate antiviral drugs require preclinical assessment of their potential for selecting resistant viruses. Although resistance data generated *in vitro* are not necessarily predictive of subsequent *in vivo* resistance when the compounds are administered to patients, *in vitro* resistance selection experiments are mandatory to assess the barrier to resistance of the investigational drug and of other compounds from the same class. These characteristics can be tested in replicon systems in hepatoma cell lines, in *in vitro* cell-free biochemical assays, and/or by means of structural studies.

### 2.1. HCV Cell Culture Systems Investigating Phenotypic Resistance

*In vitro* culture of HCV has been elusive for many years because of the lack of a robust infectable cellular system. The development of bicistronic sub-genomic HCV RNA replicons has been a major step towards the establishment of a robust cell-based system that enables the performance of reliable phenotypic testing to assess drug resistance [9,10].

#### 2.1.1. Phenotypic Assays in Replicon Systems

In replicon systems, modified forms of the HCV genome replicate at high levels in human hepatoma cells. Stably transfected replicons are capable of autonomous replication, but they are unable to support the production of infectious HCV particles [11,12]. The replicon system usually uses the HCV internal ribosome entry site (IRES) and a picornavirus IRES to direct translation from the first and second cistron, respectively. The first cistron encodes the selectable neomycin phosphotransferase gene (*neo*r), in order to select G418-resistant cells, while the second cistron translates the HCV non-structural proteins required for viral replication (NS2 to NS5B or NS3 to NS5B). For efficient replication, two major characteristics are required: the selection of adaptive mutations in the viral genome that enhance replication [11,12], and the selection of particular human hepatoma cells that are highly permissive for replicon replication. The most widely used cells are the modified hepatoma cell lines Huh7.5 and Huh7-Lunet [13]. Additional modifications can facilitate the use of replicons for phenotypic resistance testing. For example, replacement of the *neo* gene with a reporter gene such as luciferase, or the addition of this gene, allows for short-term assays in which detection of RNA replication in transiently transfected cells can be performed 48–72 h post-transfection [11,12,14].

HCV resistance pathways differ between different viral genotypes and subtypes, which display different genetic barriers to selection of resistant variants according to the drug class [15,16]. An example is the frequent selection of the R155K substitution in the NS3 protease sequence under the pressure of NS3/4A inhibitors in genotype 1a *versus* its unusual selection in genotype 1b, because of the requirement for a single-nucleotide change in genotype 1a as opposed to a 2-nucleotide change in genotype 1b. Therefore, accurate determination of phenotypic resistance must be performed with replicons of different genotypes and subtypes encountered in the clinical setting. Replicon systems were originally developed with a genotype 1b sequence [9]. Replicons of other HCV isolates, covering genotypes 1a and 2a, were subsequently obtained using the same approach. [17,18,19]. More recently, replicons from genotypes 3a, 4a [20], 5a [21] and 6a [22] have been successfully developed.

#### 2.1.2. Phenotypic Assays with Cell-Culture-Derived HCV Particles (HCVcc)

A major advance in HCV research was made when a HCV isolate from a Japanese patient replicated efficiently in cell culture [19,23]; this HCV strain, called Japanese fulminant hepatitis 1 (JFH-1), belongs to genotype 2a; it can replicate inside a human hepatoma cell line (Huh-7) and produce viruses that can infect new cells [24]. Improvement of the JFH-1 replicative system was obtained with cell-culture adaptation of the JFH1 strain [25] and by the generation of virus chimeras expanding HCV infectious culture systems to other HCV genotypes. A panel of JFH1-based intergenotypic chimeras containing core-NS2 from different genotypes [26,27] and NS5A chimeras (genotypes 1 to 7) were developed [28]. They can be used to test effectiveness of and resistance to NS5A inhibitors [29]. JFH-1 derived virus genomes with insertions of reporter genes can also be used [24,30].

Although the HCVcc system presents the advantage of covering the complete viral replication cycle compared to the replicon system, HCV replicons cover a broader range of isolates and are far more flexible for genetic manipulation. The construction of replicon chimeras in which NS3 or NS5B derive from a patient’s isolate has been effective and utilized for phenotypic resistance assays [31,32,33], while replacement of NS3 or NS5B in the JFH-1 isolate by the corresponding gene from other HCV isolates drastically altered viral replication [34].

#### 2.1.3. Assessment of Cross-Resistance

An antiviral drug may select amino acid substitutions in the target protein that confers reduced susceptibility to other antivirals from the same class. This phenomenon, referred to as cross-resistance, can be assessed by phenotypic assays. Cross-resistance can be partial, affecting a subgroup of a class (example: the V36M substitution in the NS3 protease confers resistance to linear ketoamid NS3/4A protease inhibitors, e.g., boceprevir, telaprevir) or complete (example: A156T is a major class-specific mutation that affects all NS3/4A protease inhibitors) [8].

### 2.2. Assessment of the Replication Capacity

Assessing viral replication capacity in cell culture systems is important at the preclinical stage of development and during clinical development. In general, substitutions at critical residues near the highly conserved active site of a target enzyme (*i.e.*, NS3 catalytic site or the nucleotide incorporation site of RdRp) [35] will impair enzyme function, resulting in diminished replication capacity and decreased viral fitness. In contrast, drugs that target allosteric sites, such as non-nucleoside inhibitors of RdRp or NS5A inhibitors, have little impact on viral fitness [36]. Secondary substitutions may be selected together with those conferring primary resistance because their presence on the same strain improves the viral fitness of the resistant variants, *in vitro* and/or *in vivo*. Replication capacity is calculated as the ratio of the reporter gene signal at four days post-transfection to the signal at 4 h post-transfection. The relative replication capacity of a RAV can be expressed as the percentage of the normalized reporter gene signal of the variant replicon compared with a wild-type replicon (set at 100%). In clinical practice, the impact of resistance-associated substitutions on the viral replication capacity helps predict whether these variants will be selected during treatment, whether secondary adaptive mutations will be selected, and of the likelihood of their loss after drug withdrawal [37].

### 2.3. In which Contexts Should phenOtypic Assays be Performed

Replicons and cell-culture derived HCV particles can be used to:

- Identify substitutions that confer resistance to a newly tested compound *in vitro*. Wild-type (WT) replicons (or HCVcc systems) are cultured in human hepatoma cells in the presence of increasing concentrations of the investigational compound until small colonies are formed [38,39]. These colonies are then expanded and characterized by sequence analysis to identify amino acid changes relative to the WT replicon. Cell culture selection of variants resistant to the investigational drug provides insights into the genetic barrier to resistance of the compound [36]. A drug with a low genetic barrier rapidly selects fit resistant variants bearing only one or two substitutions in the targeted viral protein. In contrast, a compound with a high genetic barrier requires multiple substitutions to select for resistance and/or a longer period of time before resistant variants acquire fitness and outgrow. Selection experiments should be repeated under high and low selective pressures, in order to determine if the same or different patterns of resistance mutations develop under these two conditions and to assess the relationship of the drug concentration with the genetic barrier to resistance.
- Phenotypically characterize the effect of substitutions identified *in vitro* or *in vivo* by means of genotypic testing. Individual or multiple candidate resistance-associated substitutions are engineered into an HCV replicon by site-directed mutagenesis. The effect of the introduced substitution(s) on drug resistance is then assessed by calculating the 50% and 90% effective concentrations (EC50 and EC90) of the mutant, compared to a wild-type control in each assay. EC50 and EC90 values are calculated either manually or by means of specific software (e.g., GraphPad Prism, (GraphPad software, San Diego, CA, USA) using the mean assay signals from multiplicate samples tested in different experiments. Drug susceptibility of a given mutant is calculated by dividing the mutant’s EC50 by the wild-type EC50; it is expressed as a “fold-change”. Assay variability may be problematic. Intra-assay variability is tested by estimating the similarity of individual EC50 and EC90 measurements performed in multiple wells of a single assay run. The standard deviation of the fold-change should be low (less than 0.3) for adequate interpretation of the assay results [40]. Inter-assay variability is assessed by performing multiple independent assay runs. The standard deviation of the mean must be three-fold or less [40]. The results must be interpreted cautiously, in the light of the clinical context. Indeed, viral variants that show low-level resistance *in vitro* may sometimes be more clinically relevant *in vivo* than variants with higher-level resistance *in vitro*.

Review articles summarizing HCV resistance profiles with different DAAs have been recently published [41,42].

### 2.4. Cell-Free *in Vitro* Assays Investigating Drug Resistance

Cell-free enzyme assays have been developed to assess susceptibility of HCV enzymes to DAAs. They have been used to test the effects of individual substitutions or complex substitution patterns on the HCV enzyme activity in the presence of the drug. In NS3/4A enzyme assays, a purified NS3 protease can be used in vitro with the Fluorescence Resonance Energy Transfer (FRET) technology that reveals the enzymatic reaction [43,44]. Briefly, the NS3/4A (or NS3 N-terminal) fragment is cloned into an *Escherichia coli* expression plasmid for protein synthesis [45]. After purification, *in vitro* protease activity is measured in the presence of different concentrations of the drug. Resistance is measured as the fold-increase in the 50% and 90% inhibitory concentrations (IC50 and IC90, respectively), which correspond to the drug concentrations that inhibit the protease function by 50% and 90%, respectively. Enzyme assays are laborious and time-consuming. A new, more accessible assay has been developed, based on a coupled *in vitro* transcription/translation system, with a total turnaround time of less than 10 h [46].

The RdRp shares a common right hand structure with other RdRps, with three main domains: fingers, palm and thumb. The enzyme catalyzes the synthesis of both positive- and negative-strand RNAs [47]. A number of biochemical tools has been developed to study HCV replication [48]. They can be used to evaluate the impact of a candidate amino-acid substitution on viral enzyme efficiency and resistance to an RdRp inhibitor. The enzyme activity of a purified RdRp can be measured, for example, by detecting the incorporation of tritiated UTP into RNA transcripts. To assess drug resistance, the inhibitor is incubated with increasing amounts of wild-type or mutated enzyme, followed by the addition of nucleoside triphosphates (NTPs) and tritiated UTP. The percentage of inhibition is calculated from the initial rates of inhibited reactions relative to that of the uninhibited control reaction. Similar to NS3/4A assays, the mean IC50 and IC90 and the standard error of the mean are calculated by nonlinear regression.

NS5A has no known enzyme activity but plays a critical role in the viral lifecycle through several molecular processes involving regulation of RdRp activity, as well as interactions with HCV proteins and numerous cellular factors [49]. NS5A inhibitors directly interact with NS5A. *In vitro* binding assays have been developed to unravel the mechanisms of resistance to NS5A inhibitors. They showed that major resistance mutations significantly reduce the affinity of NS5A for the inhibitors [50].

### 2.5. Structural Studies Investigating Drug Resistance

Structural studies are repeatedly needed to help understand viral protein functions and elucidate the mechanisms by which chemically diverse inhibitors differentially bind the wild-type and drug-resistant target proteins. Methods currently used to determine the structure of a protein and its interaction with inhibitors include X-ray crystallography, nucleic magnetic resonance (NMR) spectroscopy, and computational methods.

X-ray crystallography studies the conformational flexibility and interaction of the drug with conserved or mutated residues [51,52,53,54,55,56]. The analysis of X-ray crystallography results requires expertise and careful interpretation. If the residues involved in drug resistance are in close vicinity to the drug-binding site, it is plausible that substitutions will directly interfere with compound binding. Crystallography can be used to understand cross-resistance between drugs targeting the same viral protein and to measure the genetic barrier to resistance. For instance, structural analyses of the NS3/4A protease domain revealed that with the first-wave, first-generation protease inhibitors, only a small number of tight binding interactions with the enzyme existed, because single substitutions resulted in a significant loss of inhibition and in cross-resistance [57]. Crystal structures of ternary complexes of HCV RdRd with RNA templates, nucleotides and catalytic metal ions elucidated key molecular interactions with sofosbuvir within the active site of the enzyme. They identified the possible *in vitro* selection of the S282T substitution [53]. The crystallization process is however difficult, time-consuming and has limitations related to the type and length of the proteins analyzed. Structures are thus sometimes limited to a portion of the protein of interest (for example, domain I of the NS5A protein) [55,56].

In NMR spectroscopy, the protein is purified, placed in a strong magnetic field, and then probed with radio waves. A major advantage of NMR spectroscopy is that it provides information on proteins in solution, as opposed to those locked in a crystal, permitting structural as well as functional studies. This method is particularly well suited to the study of unstable disordered proteins such as NS5A [58,59].

Structural Bioinformatics Modeling can alternatively be used to investigate the three-dimensional structural features of the drug binding site and the impact of the different amino acid substitutions. Structural modeling analyses can be generated by software, such as PyMol [44] and/or Polyphen2 [60], which model the X-ray crystal structures of mutated NS3 or NS5B proteins using wild-type structures recovered from Protein Data Bank.

## 3. Virologic Tools Used in Clinical Research and in the Clinical Setting

In clinical research and in the clinical setting, the above-presented tools are useful to help understand the *in vivo* selection of drug-resistant viruses. However, HCV circulates within a given host in the form of mixed populations with remarkable sequence variation, referred to as viral quasispecies [5]. In such contexts, minor populations intrinsically resistant to a given drug rapidly adapt to the dynamic environmental changes created by drug administration. Viral population remodeling associated with DAA-containing treatment regimen failures has been extensively studied. Mutational pathways associated with viral resistance to different classes of DAAs with various extents of cross-resistance have been unraveled.

### 3.1. Phenotypic Resistance Testing in Clinical Research

Phenotypic characterization of candidate resistance substitutions selected in treated patients must be performed either by means of site-directed mutagenesis or by introducing longer sequences derived from the clinical isolates into a chimeric replicon backbone [33,61,62]. Whenever possible, viral gene insertion should be performed in a replicon backbone with the same genotype as the original isolate. To take into account the quasispecies distribution of HCV populations and the fact that resistant substitutions are present in mixtures of viral variant populations, phenotypic assays are best performed on a mixture of isolated clones [31]. Inability to explain virologic failure, or suspicion of nontarget region changes, may require analysis of additional regions in a subset of virologic failure samples.

### 3.2. Sequencing Tools Evaluating Resistance in Clinical Research and in the Clinical Setting (Genotypic Analysis)

Genotypic tools are available to determine the individual substitution pattern of a patient’s quasispecies at a given time point. These methods include population sequencing (also called direct sequencing) [63,64], clonal sequencing [45,65], and next-generation sequencing (NGS), currently based on deep sequencing methods [66]. The relevance of one method or the other depends on the context and aim of research. Population sequencing has limited analytical sensitivity for the detection of minor variants, *i.e*., variants present at low frequencies (≤20%). Thus, the in-depth study of the dynamics of viral variants over therapy and thereafter requires more sensitive approaches. The recent development of next-generation sequencing technologies has superseded clonal sequencing and facilitated better understanding of the genetic composition and natural evolution of viral quasispecies in the presence of antiviral drugs [66].

#### 3.2.1. Population Sequencing

The current standard method for routine genotypic analysis of HCV drug resistance is population sequencing by means of the Sanger method. Population sequencing can be easily performed on clinical samples to generate a consensus sequence, which shows with appropriate sensitivity which dominant HCV variants are present in the sample’s quasispecies. However, because of the high level of divergence between viral genotypes and subtypes, genotype-specific PCR primers must be used to ensure successful amplification of the target gene(s) (NS3, NS5A or NS5B).

#### 3.2.2. Clonal Sequencing

For many years, the study of viral quasispecies was based on the separation of individual variants by means of genetic cloning or by end-point limiting dilution (EPLD), followed by Sanger sequencing. Sequencing was then conducted on individual clones isolated from a clinical sample after the viral quasispecies has been inserted into a plasmid vector and transformed into a bacterial host. Each clonal sequence represents a unique variant population present in the mixture. However, the number of clones that can be analyzed is limited and the method is expensive and laborious. Cloning-sequencing is now being replaced by next-generation sequencing methods.

#### 3.2.3. “Next-Generation” Sequencing (NGS)

NGS refers to high-throughput sequencing technologies, which have demonstrated enormous potential in many fields of virology, including the analysis of HCV drug resistance. The currently available NGS platforms differ by their sequencing biochemistries, sequence length capacities, and throughput capabilities. The most recent techniques, such as the widely used Illumina technology, generate hundreds of millions of sequences (called “reads”) in a single run. NGS techniques are increasingly used in virology laboratories in various diagnostic applications [66,67,68,69,70,71,72,73].

NGS studies of HCV can use two strategies: whole-genome sequencing or quasispecies analysis targeting a specific gene. When focusing on short regions, NGS provides high sensitivity for the detection of minor viral populations. The “reads” are usually mapped to a genotype-specific reference sequence, such as for instance H77 in genotype 1a. The choice of the detection threshold depends on the assay variability at very low levels. Variants that represent less than 0.5% of the viral quasispecies are usually excluded, because of the risk of false-positives associated with the amplification and sequencing steps. Expertise in bioinformatics analysis of the enormous amount of reads generated with adequate computing resources is required.

### 3.3. Drug Resistance Assessment in Clinical Trials

In clinical trials, samples should be regularly collected for phenotypic and genotypic analyses, including at baseline (pre-treatment), at the time of virologic failure in case of virological breakthrough or relapse, and after treatment cessation (off-drug period follow-up). When available, the patient’s pretreatment viral sequence should serve as the reference to identify relevant changes over time. Alternatively, a standard reference sequence (such as con1, H77) can be used.

Pretreatment (baseline) assessment. Baseline samples are analyzed to detect known or unknown polymorphisms and provide a comparator for on- and post-treatment changes. Such studies showed the pre-existence, in various proportions, of RAVs conferring resistance to the known classes of DAAs. For example, the baseline frequency of RAVs conferring resistance to first-wave, first-generation NS3/4A protease inhibitors (telaprevir or boceprevir) is low (approximately 3%) when assessed by population sequencing [63,74,75]. Their presence may be predictive of treatment failure. In the telaprevir-based PROVE1/2 studies [63], patients with an R155K substitution at baseline had lower SVR rates. Pre-existing resistant variants have also been described in the NS5A and NS5B region. The presence of preexisting NS5A RAVs may have an impact on the rate of virologic response to NS5A inhibitors-containing regimen, depending on the genotype/subtype, drugs used in combination with the NS5A inhibitor and treatment duration. For example, in patients infected with genotype 3 treated with sofosbuvir and daclatasvir, post-treatment relapse was more frequent in patients with cirrhosis who harbored NS5A Y93H RAVs at baseline [76]. Similarly, in patients infected with subtype 1a treated with grazoprevir and elbavir, pre-existing NS5A RAVs conferring more than five-fold resistance *in vitro* significantly reduced the rates of virologic success [77]. The presence of variants conferring resistance to nucleotide or non-nucleoside NS5B inhibitors at baseline has not been shown to have any impact on virologic responses thus far. Resistant variants are more frequently detected with NGS because of its greater sensitivity for the detection of minor viral populations [78,79,80].

Assessment at virological failure. Samples collected at the time of virological breakthrough or relapse are analyzed to identify amino acid changes relative to baseline that confer resistance to the administered drugs. Longitudinal dynamic analysis is best performed by NGS or clonal sequencing to describe quasispecies changes. Phenotypic analysis and replication kinetics experiments can be subsequently performed to assess the level of reduced susceptibility conferred by the substitutions and evaluate their viral fitness cost.

Post-treatment assessment. Post-treatment persistence or loss of RAVs depends on the impact of the substitution on the fitness of the corresponding variant compared to the “wild-type” strain. A negative impact implies progressive decay of resistant variants in the absence of selective drug pressure. However, the wild-type virus may have been cured by therapy, so that only resistant viruses are present at relapse. In that case, RAV loss depends on the occurrence of a reversion of the resistant variant to wild type by mutation. Population sequencing is relevant if the resistant variant is present as a dominant viral population, but more sensitive techniques (*i.e*., clonal sequencing or NGS) are required to fully characterize the dynamics of RAV decay after treatment cessation. The nucleotide inhibitor sofosbuvir has a high barrier to resistance. Resistant variants are exceptionally selected and present as dominant populations at the time of relapse, because of their low *in vivo* fitness. Thus, they rapidly disappear after treatment cessation. Therefore, retreatment with a sofosbuvir-containing regimen is recommended [16]. In contrast, patients failing on regimens containing an NS3/4A protease inhibitor, a non-nucleoside RdRp inhibitor, or an NS5A inhibitor select resistant viruses that can persist for months after treatment (example: NS3/4A protease inhibitors), for years (example: non-nucleoside RdRp inhibitors), possibly for life (example: NS5A inhibitors) [81,82,83,84]. Post-treatment persistence of RAVs is also influenced by the viral genotype/subtype [81].

Reemergence of RAVs in case of a second exposure to the same drug or drug class may have clinical consequences [16,72,84]. Analyzing viral quasispecies during the off-drug period preceding retreatment is challenging. If reemergence of resistance appears with an identical genomic background, it suggests that it arises from persisting low-level RAVs selected during the first course of treatment. A different genetic background suggests *de novo* generation of resistant variants.

### 3.4. Drug Resistance Testing in Clinical Practice

HCV resistance monitoring during drug development is fundamental to understanding the clinical impact of drug resistance. In contrast, the usefulness of systematically performing HCV resistance testing in the clinical setting remains debated. In contrast to HIV infection, during which infected cells harboring resistant variants are archived for prolonged periods [85], HCV can be cured in the vast majority of cases with currently available antiviral therapies. Furthermore, depending on the drugs and amino acid substitutions, resistant variants may not have long-term clinical consequences.

HCV resistance testing prior to first-line therapy currently is not recommended [16]. Indeed, the SVR rates are very high both in patients without and with detectable amounts of pre-existing RAVs; therefore, the detection of RAVs will not influence the treatment decision. The only exception is the Q80K substitution in the protease region of HCV genotype 1a, which confers simeprevir resistance. International guidelines recommend testing for the presence of Q80K DAA-naïve patients infected with HCV subtype 1a who are being considered for treatment with simeprevir, PEG-IFN and RBV or sofosbuvir plus simeprevir in cirrhotic patients [86].

Resistance testing may be useful in patients experiencing virological breakthrough or post-treatment relapse [16], particularly when their treatment comprises NS5A inhibitors. Indeed, NS5A RAVs can remain detectable several years after treatment withdrawal [83,87]. The AASLD/IDSA guidelines recommend testing for RAVs that confer decreased susceptibility to NS3 protease and to NS5A inhibitors, for retreatment of cirrhotic patients or other patients who require retreatment urgently when these patients have history of failure to NS5A inhibitor-containing regimen [88]. In clinical practice, monitoring resistance for the persistence of RAVs will lead to better management of second-line therapy. In addition, resistance patterns derived from clinical samples are more representative of “real-life” and could differ in complexity from those observed in clinical trials, therefore reinforcing the need to perform resistance testing in the context of virological failure in the clinical setting.

## References

[1] Scheel T.K., Rice C.M. (2013). Understanding the hepatitis C virus life cycle paves the way for highly effective therapies. Nat. Med..

[2] Messina J.P., Humphreys I., Flaxman A., Brown A., Cooke G.S., Pybus O.G., Barnes E. (2015). Global distribution and prevalence of hepatitis C virus genotypes. Hepatology.

[3] Pawlotsky J.M. (2014). New hepatitis C therapies: The toolbox, strategies, and challenges. Gastroenterology.

[4] Pawlotsky J.M. (2015). Hepatitis C treatment: The data flood goes on—An update from the liver meeting 2014. Gastroenterology.

[5] Pawlotsky J.M., Germanidis G., Frainais P.O., Bouvier M., Soulier A., Pellerin M., Dhumeaux D. (1999). Evolution of the hepatitis C virus second envelope protein hypervariable region in chronically infected patients receiving α interferon therapy. J. Virol..

[6] Neumann A.U., Lam N.P., Dahari H., Gretch D.R., Wiley T.E., Layden T.J., Perelson A.S. (1998). Hepatitis C viral dynamics *in vivo* and the antiviral efficacy of interferon-α therapy. Science.

[7] Pawlotsky J.M. (2009). Hepatitis: HCV variability, the immune system and resistance to antiviral drugs. Nat. Rev. Gastroenterol. Hepatol..

[8] Pawlotsky J.M. (2011). Treatment failure and resistance with direct-acting antiviral drugs against hepatitis C virus. Hepatology.

[9] Lohmann V., Korner F., Koch J., Herian U., Theilmann L., Bartenschlager R. (1999). Replication of subgenomic hepatitis C virus RNAs in a hepatoma cell line. Science.

[10] Blight K.J., Kolykhalov A.A., Rice C.M. (2000). Efficient initiation of HCV RNA replication in cell culture. Science.

[11] Krieger N., Lohmann V., Bartenschlager R. (2001). Enhancement of hepatitis C virus RNA replication by cell culture-adaptive mutations. J. Virol..

[12] Lohmann V., Korner F., Dobierzewska A., Bartenschlager R. (2001). Mutations in hepatitis C virus RNAs conferring cell culture adaptation. J. Virol..

[13] Blight K.J., McKeating J.A., Rice C.M. (2002). Highly permissive cell lines for subgenomic and genomic hepatitis C virus RNA replication. J. Virol..

[14] Tanabe Y., Sakamoto N., Enomoto N., Kurosaki M., Ueda E., Maekawa S., Yamashiro T., Nakagawa M., Chen C.H., Kanazawa N. (2004). Synergistic inhibition of intracellular hepatitis C virus replication by combination of ribavirin and interferon α. J. Infect. Dis..

[15] Smith D.B., Bukh J., Kuiken C., Muerhoff A.S., Rice C.M., Stapleton J.T., Simmonds P. (2014). Expanded classification of hepatitis C virus into 7 genotypes and 67 subtypes: Updated criteria and genotype assignment web resource. Hepatology.

[16] European Association for the Study of the Liver (2015). EASL recommendations on treatment of Hepatitis C 2015. J. Hepatol..

[17] Blight K.J., McKeating J.A., Marcotrigiano J., Rice C.M. (2003). Efficient replication of hepatitis C virus genotype 1a RNAs in cell culture. J. Virol..

[18] Yi M., Villanueva R.A., Thomas D.L., Wakita T., Lemon S.M. (2006). Production of infectious genotype 1a hepatitis C virus (Hutchinson strain) in cultured human hepatoma cells. Proc. Natl. Acad. Sci. USA.

[19] Kato T., Date T., Miyamoto M., Furusaka A., Tokushige K., Mizokami M., Wakita T. (2003). Efficient replication of the genotype 2a hepatitis C virus subgenomic replicon. Gastroenterology.

[20] Saeed M., Scheel T.K., Gottwein J.M., Marukian S., Dustin L.B., Bukh J., Rice C.M. (2012). Efficient replication of genotype 3a and 4a hepatitis C virus replicons in human hepatoma cells. Antimicrob. Agents Chemother..

[21] Wose Kinge C.N., Espiritu C., Prabdial-Sing N., Sithebe N.P., Saeed M., Rice C.M. (2014). Hepatitis C virus genotype 5a subgenomic replicons for evaluation of direct-acting antiviral agents. Antimicrob. Agents Chemother..

[22] Yu M., Peng B., Chan K., Gong R., Yang H., Delaney W.T., Cheng G. (2014). Robust and persistent replication of the genotype 6a hepatitis C virus replicon in cell culture. Antimicrob. Agents Chemother..

[23] Kato T., Furusaka A., Miyamoto M., Date T., Yasui K., Hiramoto J., Nagayama K., Tanaka T., Wakita T. (2001). Sequence analysis of hepatitis C virus isolated from a fulminant hepatitis patient. J. Med. Virol..

[24] Wakita T., Pietschmann T., Kato T., Date T., Miyamoto M., Zhao Z., Murthy K., Habermann A., Krausslich H.G., Mizokami M. (2005). Production of infectious hepatitis C virus in tissue culture from a cloned viral genome. Nat. Med..

[25] Zhong J., Gastaminza P., Cheng G., Kapadia S., Kato T., Burton D.R., Wieland S.F., Uprichard S.L., Wakita T., Chisari F.V. (2005). Robust hepatitis C virus infection *in vitro*. Proc. Natl. Acad. Sci. USA.

[26] Gottwein J.M., Scheel T.K., Jensen T.B., Lademann J.B., Prentoe J.C., Knudsen M.L., Hoegh A.M., Bukh J. (2009). Development and characterization of hepatitis C virus genotype 1–7 cell culture systems: Role of CD81 and scavenger receptor class B type I and effect of antiviral drugs. Hepatology.

[27] Gottwein J.M., Jensen T.B., Mathiesen C.K., Meuleman P., Serre S.B., Lademann J.B., Ghanem L., Scheel T.K., Leroux-Roels G., Bukh J. (2011). Development and application of hepatitis C reporter viruses with genotype 1 to 7 core-nonstructural protein 2 (NS2) expressing fluorescent proteins or luciferase in modified JFH1 NS5A. J. Virol..

[28] Scheel T.K., Gottwein J.M., Mikkelsen L.S., Jensen T.B., Bukh J. (2011). Recombinant HCV variants with NS5A from genotypes 1–7 have different sensitivities to an NS5A inhibitor but not interferon-α. Gastroenterology.

[29] Wang C., Jia L., O’Boyle D.R., Sun J.H., Rigat K., Valera L., Nower P., Huang X., Kienzle B., Roberts S. (2014). Comparison of daclatasvir resistance barriers on NS5A from hepatitis C virus genotypes 1 to 6: Implications for cross-genotype activity. Antimicrob. Agents Chemother..

[30] Koutsoudakis G., Kaul A., Steinmann E., Kallis S., Lohmann V., Pietschmann T., Bartenschlager R. (2006). Characterization of the early steps of hepatitis C virus infection by using luciferase reporter viruses. J. Virol..

[31] Le Pogam S., Seshaadri A., Kosaka A., Chiu S., Kang H., Hu S., Rajyaguru S., Symons J., Cammack N., Najera I. (2008). Existence of hepatitis C virus NS5B variants naturally resistant to non-nucleoside, but not to nucleoside, polymerase inhibitors among untreated patients. J. Antimicrob. Chemother..

[32] Middleton T., He Y., Pilot-Matias T., Tripathi R., Lim B.H., Roth A., Chen C.M., Koev G., Ng T.I., Krishnan P. (2007). A replicon-based shuttle vector system for assessing the phenotype of HCV NS5B polymerase genes isolated from patient populations. J. Virol. Methods.

[33] Qi X., Bae A., Liu S., Yang H., Sun S.C., Harris J., Delaney W., Miller M., Mo H. (2009). Development of a replicon-based phenotypic assay for assessing the drug susceptibilities of HCV NS3 protease genes from clinical isolates. Antivir. Res..

[34] Murayama A., Date T., Morikawa K., Akazawa D., Miyamoto M., Kaga M., Ishii K., Suzuki T., Kato T., Mizokami M. (2007). The NS3 helicase and NS5B-to-3’X regions are important for efficient hepatitis C virus strain JFH-1 replication in Huh7 cells. J. Virol..

[35] Yi M., Tong X., Skelton A., Chase R., Chen T., Prongay A., Bogen S.L., Saksena A.K., Njoroge F.G., Veselenak R.L. (2006). Mutations conferring resistance to SCH6, a novel hepatitis C virus NS3/4A protease inhibitor. J. Biol. Chem..

[36] McCown M.F., Rajyaguru S., Le Pogam S., Ali S., Jiang W.R., Kang H., Symo ns J., Cammack N., Najera I. (2008). The hepatitis C virus replicon presents a higher barrier to resistance to nucleoside analogs than to nonnucleoside polymerase or protease inhibitors. Antimicrob. Agents Chemother..

[37] He Y., King M.S., Kempf D.J., Lu L., Lim H.B., Krishnan P., Kati W., Middleton T., Molla A. (2008). Relative replication capacity and selective advantage profiles of protease inhibitor-resistant hepatitis C virus (HCV) NS3 protease mutants in the HCV genotype 1b replicon system. Antimicrob. Agents Chemother..

[38] Trozzi C., Bartholomew L., Ceccacci A., Biasiol G., Pacini L., Altamura S., Narjes F., Muraglia E., Paonessa G., Koch U. (2003). *In vitro* selection and characterization of hepatitis C virus serine protease variants resistant to an active-site peptide inhibitor. J. Virol..

[39] Shih I.H., Vliegen I., Peng B., Yang H., Hebner C., Paeshuyse J., Purstinger G., Fenaux M., Tian Y., Mabery E. (2011). Mechanistic characterization of GS-9190 (Tegobuvir), a novel nonnucleoside inhibitor of hepatitis C virus NS5B polymerase. Antimicrob. Agents Chemother..

[40] Kwong A.D., Najera I., Bechtel J., Bowden S., Fitzgibbon J., Harrington P., Kempf D., Kieffer T.L., Koletzki D., Kukolj G. (2011). Sequence and phenotypic analysis for resistance monitoring in hepatitis C virus drug development: Recommendations from the HCV DRAG. Gastroenterology.

[41] Poveda E., Wyles D.L., Mena A., Pedreira J.D., Castro-Iglesias A., Cachay E. (2014). Update on hepatitis C virus resistance to –direct-acting antiviral agents. Antivir. Res..

[42] Lontok E., Harrington P., Howe A., Kieffer T., Lennerstrand J., Lenz O., McPhee F., Mo H., Parkin N., Pilot-Matias T. (2015). Hepatitis C virus drug resistance-associated substitutions: State of the art summary. Hepatology.

[43] Lin C., Lin K., Luong Y.P., Rao B.G., Wei Y.Y., Brennan D.L., Fulghum J.R., Hsiao H.M., Ma S., Maxwell J.P. (2004). In vitro resistance studies of hepatitis C virus serine protease inhibitors, VX-950 and BILN 2061: Structural analysis indicates different resistance mechanisms. J. Biol. Chem..

[44] Barbotte L., Ahmed-Belkacem A., Chevaliez S., Soulier A., Hezode C., Wajcman H., Bartels D.J., Zhou Y., Ardzinski A., Mani N. (2010). Characterization of V36C, a novel amino acid substitution conferring hepatitis C virus (HCV) resistance to telaprevir, a potent peptidomimetic inhibitor of HCV protease. Antimicrob. Agents Chemother..

[45] Sarrazin C., Kieffer T.L., Bartels D., Hanzelka B., Muh U., Welker M., Wincheringer D., Zhou Y., Chu H.M., Lin C. (2007). Dynamic hepatitis C virus genotypic and phenotypic changes in patients treated with the protease inhibitor telaprevir. Gastroenterology.

[46] Qiao J., Yu J., Yang H., Wei H. (2014). PCR-based *in vitro* synthesis of hepatitis C virus NS3 protease for rapid phenotypic resistance testing of protease inhibitors. J. Clin. Microbiol..

[47] Wang Q.M., Hockman M.A., Staschke K., Johnson R.B., Case K.A., Lu J., Parsons S., Zhang F., Rathnachalam R., Kirkegaard K. (2002). Oligomerization and cooperative RNA synthesis activity of hepatitis C virus RNA-dependent RNA polymerase. J. Virol..

[48] Liu Y., Jiang W.W., Pratt J., Rockway T., Harris K., Vasavanonda S., Tripathi R., Pithawalla R., Kati W.M. (2006). Mechanistic study of HCV polymerase inhibitors at individual steps of the polymerization reaction. Biochemistry.

[49] Pawlotsky J.M. (2013). NS5A inhibitors in the treatment of hepatitis C. J. Hepatol..

[50] Kwon H.J., Xing W., Chan K., Niedziela-Majka A., Brendza K.M., Kirschberg T., Kato D., Link J.O., Cheng G. (2015). Direct Binding of Ledipasvir to HCV NS5A: Mechanism of Resistance to an HCV Antiviral Agent. PLoS ONE.

[51] Kim J.L., Morgenstern K.A., Lin C., Fox T., Dwyer M.D., Landro J.A., Chambers S.P., Markland W., Lepre C.A., O’Malley E.T. (1996). Crystal structure of the hepatitis C virus NS3 protease domain complexed with a synthetic NS4A cofactor peptide. Cell.

[52] Lesburg C.A., Cable M.B., Ferrari E., Hong Z., Mannarino A.F., Weber P.C. (1999). Crystal structure of the RNA-dependent RNA polymerase from hepatitis C virus reveals a fully encircled active site. Nat. Struct. Biol..

[53] Appleby T.C., Perry J.K., Murakami E., Barauskas O., Feng J., Cho A., Fox D., Wetmore D.R., McGrath M.E., Ray A.S. (2015). Viral replication. Structural basis for RNA replication by the hepatitis C virus polymerase. Science.

[54] Romano K.P., Ali A., Aydin C., Soumana D., Ozen A., Deveau L.M., Silver C., Cao H., Newton A., Petropoulos C.J. (2012). The molecular basis of drug resistance against hepatitis C virus NS3/4A protease inhibitors. PLoS Pathog..

[55] Tellinghuisen T.L., Marcotrigiano J., Rice C.M. (2005). Structure of the zinc-binding domain of an essential component of the hepatitis C virus replicase. Nature.

[56] Love R.A., Brodsky O., Hickey M.J., Wells P.A., Cronin C.N. (2009). Crystal structure of a novel dimeric form of NS5A domain I protein from hepatitis C virus. J. Virol..

[57] Romano K.P., Ali A., Royer W.E., Schiffer C.A. (2010). Drug resistance against HCV NS3/4A inhibitors is defined by the balance of substrate recognition *versus* inhibitor binding. Proc. Natl. Acad. Sci. USA.

[58] Penin F., Brass V., Appel N., Ramboarina S., Montserret R., Ficheux D., Blum H.E., Bartenschlager R., Moradpour D. (2004). Structure and function of the membrane anchor domain of hepatitis C virus nonstructural protein 5A. J. Biol. Chem..

[59] Verdegem D., Badillo A., Wieruszeski J.M., Landrieu I., Leroy A., Bartenschlager R., Penin F., Lippens G., Hanoulle X. (2011). Domain 3 of NS5A protein from the hepatitis C virus has intrinsic α-helical propensity and is a substrate of cyclophilin A. J. Biol. Chem..

[60] Polyphen2. http://genetics.bwh.harvard.edu/pph2.

[61] Tripathi R.L., Krishnan P., He Y., Middleton T., Pilot-Matias T., Chen C.M., Lau D.T., Lemon S.M., Mo H., Kati W. (2007). Replication efficiency of chimeric replicon containing *NS5A-5B* genes derived from HCV-infected patient sera. Antivir. Res..

[62] Kati W., Koev G., Irvin M., Beyer J., Liu Y., Krishnan P., Reisch T., Mondal R., Wagner R., Molla A. (2015). *In vitro* activity and resistance profile of dasabuvir, a nonnucleoside hepatitis C virus polymerase inhibitor. Antimicrob. Agents Chemother..

[63] Bartels D.J., Zhou Y., Zhang E.Z., Marcial M., Byrn R.A., Pfeiffer T., Tigges A.M., Adiwijaya B.S., Lin C., Kwong A.D. (2008). Natural prevalence of hepatitis C virus variants with decreased sensitivity to NS3/4A protease inhibitors in treatment-naive subjects. J. Infect. Dis..

[64] Kieffer T.L., Kwong A.D., Picchio G.R. (2010). Viral resistance to specifically targeted antiviral therapies for hepatitis C (STAT-Cs). J. Antimicrob. Chemother..

[65] Pawlotsky J.M., Germanidis G., Neumann A.U., Pellerin M., Frainais P.O., Dhumeaux D. (1998). Interferon resistance of hepatitis C virus genotype 1b: Relationship to nonstructural *5A* gene quasispecies mutations. J. Virol..

[66] Chevaliez S., Rodriguez C., Pawlotsky J.M. (2012). New virologic tools for management of chronic hepatitis B and C. Gastroenterology.

[67] Hiraga N., Imamura M., Abe H., Hayes C.N., Kono T., Onishi M., Tsuge M., Takahashi S., Ochi H., Iwao E. (2011). Rapid emergence of telaprevir resistant hepatitis C virus strain from wildtype clone *in vivo*. Hepatology.

[68] Verbinnen T., van Marck H., Vandenbroucke I., Vijgen L., Claes M., Lin T.I., Simmen K., Neyts J., Fanning G., Lenz O. (2010). Tracking the evolution of multiple *in vitro* hepatitis C virus replicon variants under protease inhibitor selection pressure by 454 deep sequencing. J. Virol..

[69] Wang G.P., Sherrill-Mix S.A., Chang K.M., Quince C., Bushman F.D. (2010). Hepatitis C virus transmission bottlenecks analyzed by deep sequencing. J. Virol..

[70] Donaldson E.F., Harrington P.R., O'Rear J.J., Naeger L.K. (2015). Clinical evidence and bioinformatics characterization of potential hepatitis C virus resistance pathways for sofosbuvir. Hepatology.

[71] Sato M., Maekawa S., Komatsu N., Tatsumi A., Miura M., Muraoka M., Suzuki Y., Amemiya F., Takano S., Fukasawa M. (2015). Deep Sequencing and Phylogenetic Analysis of Variants Resistant to Interferon-Based Protease Inhibitor Therapy in Chronic Hepatitis Induced by Genotype-1b Hepatitis C Virus. J. Virol..

[72] Susser S., Flinders M., Reesink H.W., Zeuzem S., Lawyer G., Ghys A., van Eygen V., Witek J., de Meyer S., Sarrazin C. (2015). Evolution of Hepatitis C virus quasispecies during repeated treatment with the NS3/4A protease inhibitor telaprevir. Antimicrob. Agents Chemother..

[73] Ho C.K., Welkers M.R., Thomas X.V., Sullivan J.C., Kieffer T.L., Reesink H.W., Rebers S.P., de Jong M.D., Schinkel J., Molenkamp R. (2015). A comparison of 454 sequencing and clonal sequencing for the characterization of hepatitis C virus NS3 variants. J. Virol. Methods.

[74] Kuntzen T., Timm J., Berical A., Lennon N., Berlin A.M., Young S.K., Lee B., Heckerman D., Carlson J., Reyor L.L. (2008). Naturally occurring dominant resistance mutations to hepatitis C virus protease and polymerase inhibitors in treatment-naive patients. Hepatology.

[75] Colson P., Brouk N., Lembo F., Castellani P., Tamalet C., Gerolami R. (2008). Natural presence of substitution R155K within hepatitis C virus NS3 protease from a treatment-naive chronically infected patient. Hepatology.

[76] Nelson D.R., Cooper J.N., Lalezari J.P., Lawitz E., Pockros P.J., Gitlin N., Freilich B.F., Younes Z.H., Harlan W., Ghalib R. (2015). All-oral 12-week treatment with daclatasvir plus sofosbuvir in patients with hepatitis C virus genotype 3 infection: ALLY-3 phase III study. Hepatology.

[77] Zeuzem S., Rockstroh J.K., Kwo P.Y., Roth D., Lawitz E., Sulkowski M.S., Forns X., Wahl J., Robertson M., Nguyen B.T. Predictors of Response to Grazoprevir/Elbasvir among HCV Genotype 1 (GT1)-Infected Patients: Integrated Analysis of Phase 2–3 Trials. Proceeding of the AASLD Liver Meeting.

[78] Fonseca-Coronado S., Escobar-Gutierrez A., Ruiz-Tovar K., Cruz-Rivera M.Y., Rivera-Osorio P., Vazquez-Pichardo M., Carpio-Pedroza J.C., Ruiz-Pacheco J.A., Cazares F., Vaughan G. (2012). Specific detection of naturally occurring hepatitis C virus mutants with resistance to telaprevir and boceprevir (protease inhibitors) among treatment-naive infected individuals. J. Clin. Microbiol..

[79] Lauck M., Alvarado-Mora M.V., Becker E.A., Bhattacharya D., Striker R., Hughes A.L., Carrilho F.J., O’Connor D.H., Pinho J.R. (2012). Analysis of hepatitis C virus intrahost diversity across the coding region by ultradeep pyrosequencing. J. Virol..

[80] Nasu A., Marusawa H., Ueda Y., Nishijima N., Takahashi K., Osaki Y., Yamashita Y., Inokuma T., Tamada T., Fujiwara T. (2011). Genetic heterogeneity of hepatitis C virus in association with antiviral therapy determined by ultra-deep sequencing. PLoS ONE.

[81] Sullivan J.C., de Meyer S., Bartels D.J., Dierynck I., Zhang E.Z., Spanks J., Tigges A.M., Ghys A., Dorrian J., Adda N. (2013). Evolution of treatment-emergent resistant variants in telaprevir phase 3 clinical trials. Clin. Infect. Dis..

[82] Wang C., Sun J.H., O’Boyle D.R., Nower P., Valera L., Roberts S., Fridell R.A., Gao M. (2013). Persistence of resistant variants in hepatitis C virus-infected patients treated with the NS5A replication complex inhibitor daclatasvir. Antimicrob. Agents Chemother..

[83] Krishnan P., Tripathi R., Schnell G., Reisch T., Beyer J., Dekhtyar T., Irvin M., Xie W., Larsen L., Podsadecki T. (2015). Long-term follow-up of treatment-emergent resistance-associated variants in NS3, NS5A and NS5B with paritaprevir/r, Ombitasvir and Dasabuvir-based regimens. J. Hepatol..

[84] Akuta N., Suzuki F., Sezaki H., Suzuki Y., Hosaka T., Kobayashi M., Saitoh S., Ikeda K., Kumada H. (2014). Evolution of simeprevir-resistant variants over time by ultra-deep sequencing in HCV genotype 1b. J. Med. Virol..

[85] Finzi D., Blankson J., Siliciano J.D., Margolick J.B., Chadwick K., Pierson T., Smith K., Lisziewicz J., Lori F., Flexner C. (1999). Latent infection of CD4^+^ T cells provides a mechanism for lifelong persistence of HIV-1, even in patients on effective combination therapy. Nat. Med..

[86] Lawitz E., Matusow G., DeJesus E., Yoshida E., Felizarta F., Ghalib R., Godofsky E., Herring R., Poleynard G., Sheikh A. A phase 3, open-label, single-arm study to evaluate the efficacy and safety of 12 weeks of simeprevir (SMV) plus sofosbuvir (SOF) in treatment-naive or -experienced patients with chronic HCV genotype 1 infection and cirrhosis: OPTIMIST-2. Proceedings of the 50th Annual Meeting of the European Association for the Study of the Liver (EASL).

[87] Wyles D., Mangia A., Cheng W., Shafran S., Schwabe C., Ouyang W., Chodavarapu K., McNally J., Doehle B., Svarovskaia E. Long-Term Persistence of HCV NS5A Variants After Treatment With NS5A Inhibitor Ledipasvir. Proceeding of The International Liver Congress on 50th annual Meeting of the European association for the Study of the Liver (EASL).

[88] AASLD/IDSA HCV Guidance Panel (2015). Hepatitis C Guidance: AASLD-IDSA Recommendations for Testing, Managing, and Treating Adults Infected With Hepatitis C Virus. Hepatology.

